# A Novel Coronavirus Disease (COVID-19): a Review of Host Cell Signaling Pathways

**Published:** 2020-11

**Authors:** Hadiseh Mohammadpour, Ali Ziai, Makan Sadr, Mitra Rezaei, Majid Marjani, Payam Tabarsi

**Affiliations:** 1 Dental Research Center, Dentistry Research Institute, Tehran University of Medical Sciences, Tehran, Iran,; 2 Department of Pharmacology, School of Medicine, Shahid Beheshti University of Medical Sciences, Tehran, Iran,; 3 Virology Research Center, National Research Institutes of Tuberculosis and Lung Diseases (NRITLD), Shahid Beheshti University of Medical Sciences, Tehran, Iran,; 4 Clinical Tuberculosis and Epidemiology Research Center, NRITLD, Shahid Beheshti University of Medical Sciences, Tehran, Iran

**Keywords:** Coronavirus, Host-pathogen interactions, Signaling pathways

## Abstract

Coronaviruses (CoVs) are the largest group of positive-sense RNA viruses. By increasing our understanding of the interactions between CoVs and the host innate immune system, we can evaluate the development and persistence of inflammation in the lungs and reduce the risk of CoV-induced lung inflammation with a new group of genetic variants. Here, we aim to discuss some recent changes in host cell factors that may be used by CoV to promote the proliferation cycle. We also discuss different host cell signaling pathways that can be considered in the host-pathogen interactions at the molecular level. The pandemic of coronavirus disease 2019 (COVID-19), caused by severe acute respiratory syndrome coronavirus 2 (SARS-CoV-2), has created new challenges for the cultural, economic, and health infrastructures. Therefore, it is important that healthcare systems and physicians recognize a global integrated framework for monitoring the progression of COVID-19 to develop targeted therapies that can potentially save human lives.

## INTRODUCTION

Coronaviruses (CoVs) are single-stranded, positive-sense RNA viruses that are widely distributed in humans and other mammals, causing respiratory, gastrointestinal and neurological disorders (1).

## GENOMIC STRUCTURE & MORPHOLOGY

Under electron microscopy, CoVs appear to be almost spherical or relatively polymorphic, with distinct “club-like” predictions formed by the spike protein (S) (2, 3). There is a symmetric nucleocapsid inside the virion, which is a helical positive-sense RNA virus genome with an extremely large size (about 26–32 kb) (4). The positive-sense RNA viral genome acts as a messenger RNA (mRNA), with a 5’-terminal helical structure and a 3’poly (A) tail. This genomic RNA has three functions in the virus cycle including: RNA of the viral infection cycle; a template for replication and transcription; and a packaging substrate for the offspring (5).

## CLINICAL FEATURES

The symptoms of coronavirus disease 2019 (COVID-19) are non-specific. The most common symptoms include fever, general malaise, and dry cough. Some patients experience headaches or myalgia, although upper respiratory symptoms, such as runny nose, are not common (6). More than half of patients with COVID-19 experience shortness of breath. The median time from the onset of disease to shortness of breath is about eight days (7). Patients with COVID-19 may progress to acute respiratory distress syndrome (ARDS), followed by septic shock, refractory metabolic acidosis, and coagulation dysfunction if the infection is not controlled (6).

A minority of patients with COVID-19 progress to the most severe stage of disease, that is, extrapulmonary systemic hyperinflammatory syndrome, when the markers of systemic inflammation appear to be rising (8). Therefore, inflammatory cytokines and biomarkers, such as interleukins (IL-2, IL-6, and IL-7), granulocyte colony-stimulating factors, macrophage inflammatory protein-1α, tumor necrosis factor-α (TNF-α), C-reactive protein, ferritin, and D-dimer are elevated in patients with more severe COVID-19 (9).

## HOST-COV INTERACTIONS & SIGNALING PATHWAYS

CoVs tend to bind to angiotensin-converting enzyme 2 (ACE2). The renin-angiotensin system (RAS) is a signaling pathway for homeostatic regulation. The ACE enzyme is a zinc-dependent metallopeptidase with kinase activity by removing histidine and leucine from the C-terminal of ACE, converting angiotensin I (AngI) to angiotensin II (AngII). Overall, ACE and AngII are major contributors to clinical problems, especially ARDS in patients. ACE2 is a regulator of AngII conversion to Ang-[1–7], and ACE2 contains only one active site. ACE2 can also convert AngI to Ang-[1–9] and finally activate Mas-related G protein– coupled receptors.

ACE2 and transmembrane protease, serine 2 (TMPRSS2) are localized on cell surfaces, and CoVs use them to enter the cells. It has been shown that S protein binds to the catalytic domain of ACE2 with high affinity (10–12). Toll-like receptor 4 (TLR4) identifies S protein and activates the inflammatory pathway through MYD88 (13). TLRs have a conserved 200-amino-acid cytoplasmic domain, called the Toll/IL-1 receptor domain (TIR), which binds to and shares homology with IL-1 receptors. The MyD88 pathway is dependent on the activation of TLR/IL-1R signaling after binding to ligands. MyD88 forms a complex with IL-1 receptor-associated kinase (IRAK) and TNF receptor–associated factor (TRAF) family members. On the other hand, TRAF binds to IRAK and activates TAK1 through IRAK1 phosphorylation. TAK1 is utilized by TLR and activates NF-κB and MAPK signaling pathways for inflammatory cytokines, and the related genes are induced in an immune response to viral infection.

The NF-κB pathway is activated by TAK1, which phosphorylates another protein kinase, called the IκB kinase (IKK) complex. The IKB7 protein inhibits NF-κB through phosphorylation of IKK by IκB. Free NF-κB activates and enters the nucleus, and NF-κB activation of transcription factors finally produces cytokines through immune cells. TAK1 phosphorylates and activates mitogen-activated protein kinase (MAPK) and leads to c-Jun N-terminal kinase (JNK) phosphorylation of p53 tumor suppressor (14, 15). The TLR-MyD88 complex stimulates T cells that regulate adaptive Th1 and Th2 responses. Th1 secretes IFN-1 and TNF-β, which in turn activate macrophages and also induce IgG immunoglobulin; also, Th2 produces IL-5, IL-4, IL-10, and IL-13 (16, 17).

The stimulator of interferon gene (STING) and TLR complex pathways are dependent on IL-1 and type I interferon (IFN) (18). The conserved PLPLRT/SD sequence motif at the C-terminus of STING intercedes the activation of phosphorylation by TBK1. Finally, STING produces and releases interferons (IFNs), which contribute to immune responses to viral infections (19). STING interacts with TANK binding kinase 1 (TBK1) and interferon regulatory factor-3 (IRF-3). TBK1 can affect humoral immune responses by production and signaling of type I IFN (20). STAT1 and STAT2 are activated by type I, II, and III IFN, while STAT4 and STAT6 are dependent on Th1 and Th2. STAT proteins are required for cellular differentiation of immune responses to viral infections; these proteins are also involved in cytokine signals (21). Cytokines, such as TNF-α, IL-6, and IL-1, can contribute to inflammation (20).

Moreover, the Janus kinase/signal transducers and activators of transcription (JAK-STAT) signaling pathway is involved in inflammatory and autoimmune diseases (22). Also, SOCS-1 and SOCS3 play regulatory roles in both Th1 and Th2 responses. The cytokine signaling pathway is regulated by various systems and mechanisms, including suppressors of cytokine signaling (SOCS) proteins, which suppress signaling to JAK or cytokine receptors. SOCS1 and SOCS3 are also inhibitors of JAK signaling pathway through activating their kinase inhibitory region (23).

**Figure 1.**
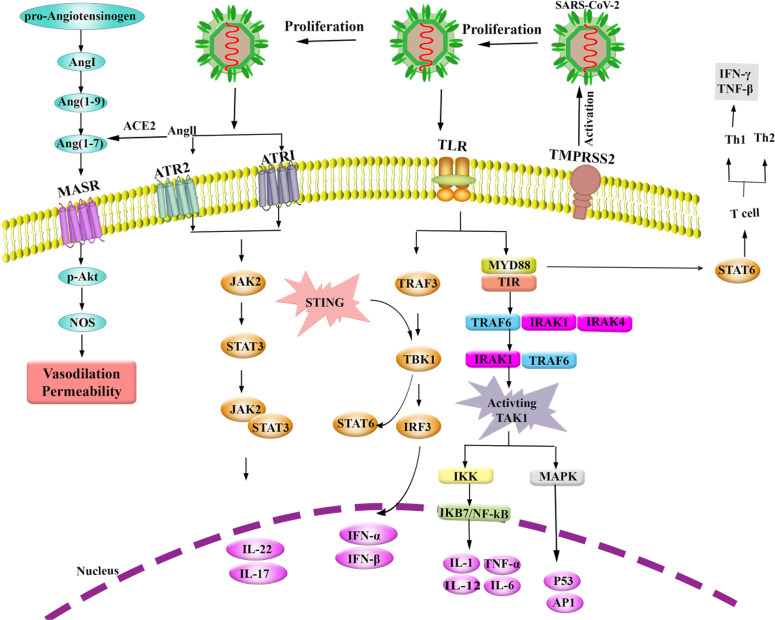
Interactions between SARS-CoV-2 and host cell; proposed signaling pathways **Abbreviations:** ***Ang:*** angiotensin; ***MASR:*** Mas-related G protein-coupled receptor; ***ACE:*** angiotensin-converting enzyme; ***ATR1 & 2:*** angiotensin type 1 & 2 receptors; ***JAK:*** Janus kinase; ***STAT:*** signal transducers and activators of transcription; ***p-Akt:*** phosphoprotein kinase B; ***NOS:*** nitric oxide synthase; ***STING:*** stimulator of interferon gene; ***TLR:*** Toll-like receptor; ***TRAF:*** TNF receptor-associated factor; ***TBK:*** TANK binding kinase; ***IRF:*** interferon regulatory factor; ***MYD88:*** Myeloid differentiation primary response 88; ***TIR:*** Toll/IL-1 receptor domain; ***IRAK:*** IL-1 receptor-associated kinase; ***IKK:*** IκB kinase; ***MAPK:*** mitogen-activated protein kinase; ***JKB:*** inhibitor of κB; ***NF-kB:*** nuclear factor-κB; ***TMPRSS2:*** transmembrane protease serine 2; ***IFN:*** Interferon; ***TNF:*** Tumor necrosis factor; ***Th:*** T helper; ***IL:*** Interleukin; ***P53:*** protein 53; ***API:*** Apoptosis inhibitors protein.

## CONCLUSION

Since the severe stage of COVID-19 is associated with host immune responses and systemic hyperinflammation of the virus-activated signaling pathway, better understanding of this pathway and cytokine production can help us develop a targeted therapy for this disease. At this stage, the use of cytokine inhibitors, such as tocilizumab (IL-6 inhibitor) or anakinra (IL-1 receptor antagonist), as well as intravenous immune globulin (IVIG) and JAK inhibitors, may modulate the immune system in an anti-inflammatory state. Overall, the recovery and prognosis of the critical stage of COVID-19 are poor, and rapid diagnosis and establishment of treatment may be effective.
